# Phosphorylation of Thr-225 and Ser-262 on ERD7 Promotes Age-Dependent and Stress-Induced Leaf Senescence through the Regulation of Hydrogen Peroxide Accumulation in *Arabidopsis thaliana*

**DOI:** 10.3390/ijms25021328

**Published:** 2024-01-22

**Authors:** Rongrong Wu, Xiaolu Pan, Wei Li, Zenglin Zhang, Yongfeng Guo

**Affiliations:** 1 College of Agriculture, Qingdao Agricultural University, Qingdao 266000, China; wurongrong515@163.com; 2 Tobacco Research Institute, Chinese Academy of Agricultural Sciences, Qingdao 266000, China; pxlat4820@163.com (X.P.); liwei06@caas.cn (W.L.)

**Keywords:** ERD7, leaf senescence, phosphorylation, ROS, *Arabidopsis thaliana*

## Abstract

As the final stage of leaf development, leaf senescence is affected by a variety of internal and external signals including age and environmental stresses. Although significant progress has been made in elucidating the mechanisms of age-dependent leaf senescence, it is not clear how stress conditions induce a similar process. Here, we report the roles of a stress-responsive and senescence-induced gene, *ERD7* (*EARLY RESPONSIVE TO DEHYDRATION 7*), in regulating both age-dependent and stress-induced leaf senescence in Arabidopsis. The results showed that the leaves of *erd7* mutant exhibited a significant delay in both age-dependent and stress-induced senescence, while transgenic plants overexpressing the gene exhibited an obvious accelerated leaf senescence. Furthermore, based on the results of LC-MS/MS and PRM quantitative analyses, we selected two phosphorylation sites, Thr-225 and Ser-262, which have a higher abundance during senescence, and demonstrated that they play a key role in the function of ERD7 in regulating senescence. Transgenic plants overexpressing the phospho-mimetic mutant of the activation segment residues ERD7^T225D^ and ERD7^T262D^ exhibited a significantly early senescence, while the inactivation segment ERD7^T225A^ and ERD7^T262A^ displayed a delayed senescence. Moreover, we found that ERD7 regulates ROS accumulation by enhancing the expression of *AtrbohD* and *AtrbohF,* which is dependent on the critical residues, i.e., Thr-225 and Ser-262. Our findings suggest that ERD7 is a positive regulator of senescence, which might function as a crosstalk hub between age-dependent and stress-induced leaf senescence.

## 1. Introduction

Leaf senescence, a process of programmed cell apoptosis, is regulated by developmental programs integrated with a variety of endogenous and exogenous factors including hormones and stresses [1,2]. During senescence, the metabolic processes in a leaf undergo dramatic changes. Macromolecules in senescing leaves, including proteins, nucleic acids, carbohydrates, and lipids, are degraded, and the resulting metabolic products are transported to sink tissues such as new leaves and reproductive organs for nutrient recycling [1,3,4]. In agricultural production, abiotic stresses and pathogen infections often cause the premature senescence of crop plants, leading to a significant yield reduction [3]. Therefore, the identification and characterization of leaf senescence regulator is conducive to crop yield increase under optimum and adverse growth conditions [5].

Age-dependent senescence is closely connected with the regulatory pathways of endogenous phytohormones and exogenous environmental stresses [6]. When plants are under adverse environmental conditions, the growth and development will be affected, often resulting in premature senescence as a form of adaptability [7,8]. It has been shown that environmental factors, such as darkness, nutrient restriction, drought, heat or cold, high salinity, and pathogen attack or injury, could trigger leaf senescence [9,10]. Salt stress is one of the major promoters of plant senescence [11]. Plants grown in a high-salinity environment will have stress responses and often show early senescence, which often reduce crop yield [2]. Plant hormones interact with stress responses through a complex network of signaling pathways. It has been reported that abscisic acid (ABA), ethylene (ETH), brassinosteroid (BR), jasmonic acid (JA), and salicylic acid (SA) have the effect of promoting senescence, while cytokinins (CK), gibberellic acid (GA), and auxin (IAA) have the effect of inhibiting senescence [12,13]. ABA is one of the key hormones in plant response to environmental stresses [14]. Leaf senescence induced by drought, high salinity, and cold stresses can promote the accumulation of endogenous ABA and the expression of ABA signaling-related genes [6,15]. Furthermore, ABA plays a positive role in regulating leaf senescence of different plant species such as *Arabidopsis* and rice, suggesting that ABA may play a conservative role in leaf senescence and may be a hormonal trigger of stress-induced senescence [16]. 

Extensive studies have demonstrated that age-dependent changes in reactive oxygen species (ROS) levels are essential for the progression of leaf senescence [11]. ROS in cells mainly include hydrogen peroxide (H_2_O_2_), superoxide anion (O^2•−^), hydroxyl radical (HO^•^), and other signaling molecules and toxic molecules. H_2_O_2_ has been extensively studied as a relatively stable ROS signal that increases during the developmental transition to bolting [11]. Normally, the high concentrations of ROS will lead to the oxidative damage of proteins, lipids, nucleic acids, and other molecules in cells, which is a typical phenomenon during senescence [1,17]. Plants often show an increased accumulation of ROS as they undergo developmentally regulated senescence [18]. In addition, a variety of abiotic and biological stresses are known to enhance ROS accumulation [19]. In plants, the respiratory burst oxidase homologs (Rbohs) are ROS-producing enzymes that regulate plant growth and development and response to abiotic stresses [20]. AtRbohD and AtRbohF are closely related to ROS production in ABA signaling, and the AtRbohD-dependent ROS production can increase a plant’s tolerance to stresses [21]. Therefore, ROS levels seem to be a common signal in age-dependent and stress-induced senescence [18,22].

At the transcription level, leaf senescence is coordinated by multiple gene regulatory networks, a subset of which are known as senescence-associated genes (SAGs) [7]. It has been documented that the expression pattern of SAGs is also affected by environmental stresses. Previous studies have shown that the ERD genes are responsive to a number of adversity stress signals such as drought, cold, high salt, and ABA [23]. Several ERDs are commonly used as marker genes of plant stress responses [24]. In *Arabidopsis thaliana*, a total of 16 ERD genes were identified, among which ERD7 contains a senescent structural domain with lipid-binding activity and belongs to the Arabidopsis SEN family of proteins, which, as a plant-specific gene, is highly conserved across different plant species [25,26]. The C-terminus of the ERD7 protein contains a senescence structural domain [27], which consists of about 450 amino acid residues, and has been reported to be associated with senescence in several plant species [27,28,29].

In this study, we demonstrated that *ERD7*, a stress-responsive and senescence induced gene in Arabidopsis, was involved in age-dependent and stress-induced leaf senescence. Additionally, through the Parallel Reaction Monitoring (PRM) strategy [30,31,32,33], we identified two phosphorylation sites, Thr-225 and Ser-262, the phosphorylation of which was up-regulated during leaf senescence and was essential for the function of ERD7 in regulating senescence. Furthermore, ERD7 could affect cell H_2_O_2_ balance by regulating two NADPH oxidase-encoding genes, *AtrbohD* and *AtrbohF,* depending on the phosphorylation modification of Thr-225 and Ser-262. Collectively, our results reveal a novel regulatory mechanism in which ERD7 regulates ROS accumulation depending on the phosphorylation of the Thr-225 and Ser-262 residues, resulting in a fine-tuned regulation of both age-dependent and stress-induced leaf senescence. Therefore, our study of ERD7 will undoubtedly enrich the function of ERD genes, which may play an important role in the regulation of leaf senescence in addition to serving as a marker of plant stress response.

## 2. Results

### 2.1. ERD7 Expression Is Associated with Leaf Senescence and Can Be Induced by Multiple Stresses

**Figure 1:**
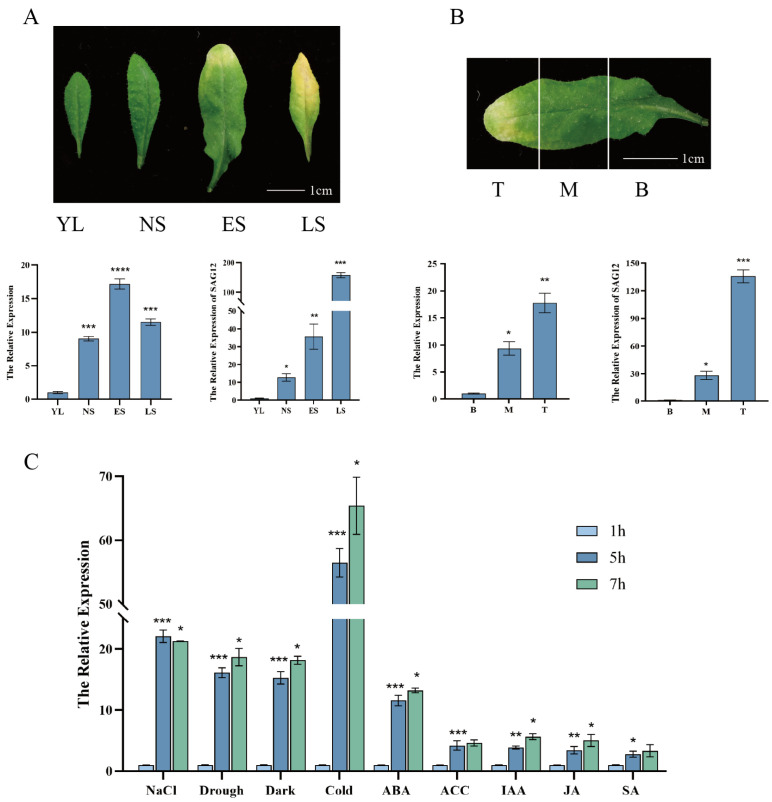
*ERD7* expression is associated with leaf senescence and multiple stresses. (**A**) Four periods of leaf growth and development in *Arabidopsis thaliana*: YL, young leaf; NS, non-senescent leaf; ES, early senescent leaf; and LS, late senescent leaf. (**B**) Three parts of senescent leaves: T, top; M, middle; and B, base. (**C**) Effect of different stress treatments on *ERD7* expression. Asterisks indicate statistically significant differences (* *p* < 0.05; ** *p* < 0.01; *** *p* < 0.001,**** *p* < 0.0001) using a Student’s *t*-test. Three independent experiments were performed.

To investigate the potential function of ERD7 in both leaf senescence and stress response, we examined the expression pattern of *ERD7*. The qRT-qPCR results showed that *ERD7* expression increased during the leaf senescence process, reaching the highest level at the ES (early senescence) stage, then decreased at the LS (late senescence) stage in the 7th rosette leaves of 5-week-old Arabidopsis plants (Figure 1A). In Arabidopsis, senescence proceeds from the leaf tip toward the leaf base. The *ERD7* transcripts in different parts of ES leaves were detected using qRT-PCR, and the results showed that the expression level of *ERD7* was higher in the tip and lower in the base of a leaf (Figure 1B). The expression pattern of *ERD7* was similar to that of the senescence marker gene *SAG12* (Figure 1A,B), suggesting that ERD7 may be involved in the regulation of leaf senescence. Additionally, to further investigate whether ERD7 is involved in stress response, we examined the expression levels of *ERD7* after treatments with different hormones and stress stimuli as indicated in Figure 1C. The qRT-PCR results showed that *ERD7* expression was significantly up-regulated in response to a variety of phytohormones including ABA, JA, SA, ACC, and IAA, with the most sensitive response to ABA treatments. In addition, *ERD7* expression levels were also significantly elevated by cold, NaCl, dark, and drought treatments (Figure 1C). These results suggest that ERD7 could play a role in regulating stress-induced senescence.

### 2.2. ERD7 Plays a Positive Role in Leaf Senescence Regulation

**Figure 2:**
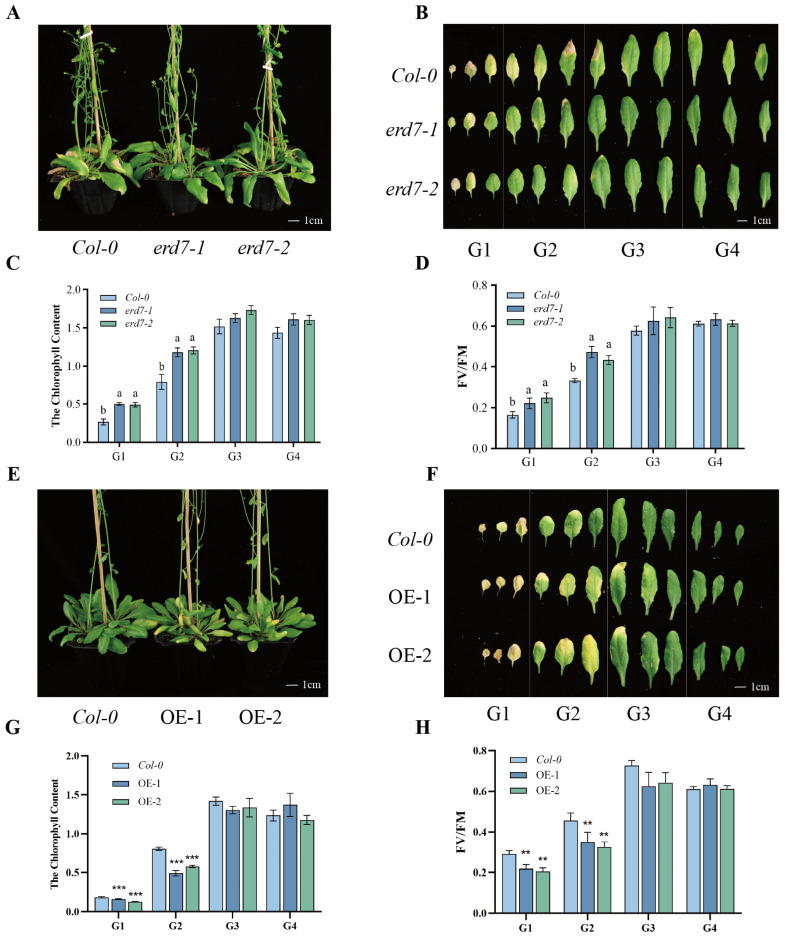
ERD7 plays positive roles in regulating age-dependent leaf senescence. (**A**) Leaf phenotypes of 6-week-old Col-0 and erd7 mutant plants. (**B**) Phenotypes of 12 rosette leaves of Col-0 and erd7 mutants. (**C**,**D**) Measurements of the chlorophyll content and the photosynthetic rate of rosette leaves of three strains. (**E**) Leaf phenotypes of 6-week-old Col-0 and *ERD7* overexpression plants. (**F**) Phenotypes of 12 rosette leaves of Col-0 and *ERD7* overexpression plants. (**G**,**H**) Measurements of the chlorophyll content and the photosynthetic rate in rosette leaves of three strains. Asterisks indicate statistically significant differences (** *p* < 0.01; *** *p* < 0.001) using a Student’s *t*-test. Different letters above columns indicate significant differences based on Duncan’s multiple range test (*p* < 0.05). Three independent experiments were performed.

**Figure 3:**
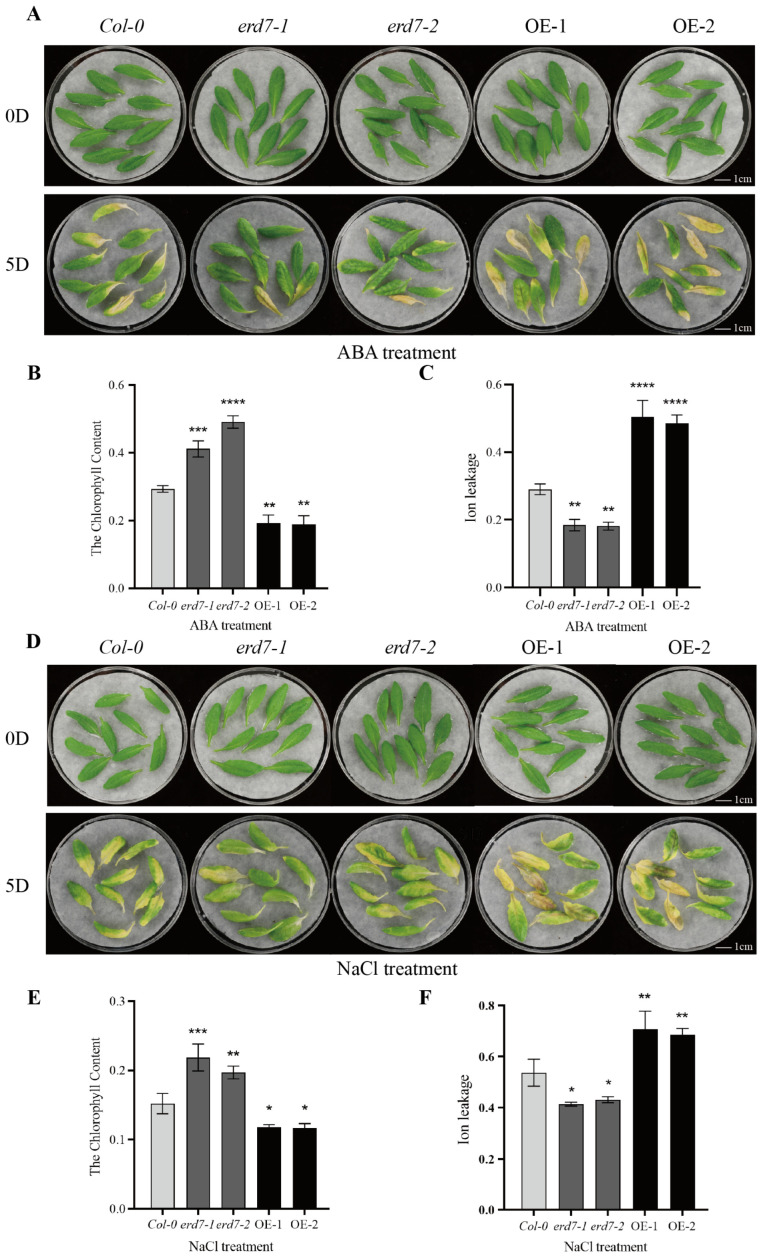
ERD7 was involved in ABA- and NaCl-induced leaf senescence. (**A**) Phenotypes of isolated leaves of *Col-0*, erd7 mutant, and *ERD7* overexpression plants after 5 μM ABA treatment. (**B**,**C**) Chlorophyll content and ion leakage measurements of isolated leaves of Col-0, erd7 mutant, and *ERD7* overexpression plants after 5 d of ABA treatment. (**D**) Phenotypes of isolated leaves of *Col-0*, erd7 mutant, and *ERD7* overexpression plants after 100 mM NaCl treatment. (**E**,**F**) Chlorophyll content and ion leakage measurements of isolated leaves of Col-0, erd7 mutant, and *ERD7* overexpression plants after 5 d of salt stress treatment. Asterisks indicate statistically significant differences (* *p* < 0.05; ** *p* < 0.01; *** *p* < 0.001; **** *p* < 0.0001) using a Student’s *t*-test. Three independent experiments were performed.

In order to further investigate the potential roles of ERD7 in leaf senescence, we obtained two independent T-DNA insertion mutant lines of the *ERD7* gene, named *erd7-1* and *erd7-2*, respectively. The qRT-PCR results showed that the expression levels of *ERD7* gene were significantly reduced in *erd7-1* and *erd7-2* when compared with those of the wild type (Figure S1), indicating that both *erd7-1* and *erd7-2* were knock-down mutants. To observe the phenotype of *erd7-1* and *erd7-2* mutants, we compared the growth and development of *erd7-1*, *erd7-2* mutants, and Col-0 wild-type. The results showed that there was no significant difference between the wild-type and the two mutants until 5 weeks after germination. Starting from 6 weeks after germination, in Col-0 plants, the signs of senescence were apparent in the older leaves. However, no senescence symptoms were observed in either *erd7-1-* or *erd7-2-*mutant plants (Figure 1A). For a better comparison of senescence phenotypes, rosette leaves from different genotypes were divided into four groups according to leaf positions (Leaf 1st–3rd: G1 group, Leaf 4th–6th: G2 group, Leaf 7th–9th: G3 group, and Leaf 10th–12th: G4 group). Compared with Col-0, leaves from the G1 and G2 groups in *erd7-1* and *erd7-2* mutants displayed a delayed senescence (Figure 2B). Leaf senescence phenotype also was assessed by measuring the typical senescence-associated physiological parameters including the chlorophyll content and the photochemical efficiency of photosystem II (PSII) Fv/Fm. In line with the visible phenotypes, G1 and G2 leaves of mutant plants had significantly higher chlorophyll concentration and Fv/Fm values (Figure 3C,D).

To further confirm the function of ERD7 in regulating leaf senescence, we generated transgenic lines overexpressing *ERD7* under the control of the *CaMV35S* promoter in Col-0 background. Two independent overexpression lines *ERD7*-OE-1 (OE-1) and *ERD7*-OE-2(OE-2) that displayed a significantly higher expression level of *ERD7* were selected for further studies (Figure S2). Six-week-old OE-1 and OE-2 plants displayed significantly accelerated leaf senescence phenotypes when compared with those of *Col-0* (Figure 2E). The senescence phenotypes from G1, G2, and G3 leaves were observed much earlier in OE1 and OE2 plants in Col-0 (Figure 2F). Furthermore, the chlorophyll contents and the Fv/Fm values of G1 and G2 group leaves were significantly lower in the overexpression lines when compared with those of Col-0 (Figure 2G,H). These results indicate that ERD7 positively regulates age-dependent leaf senescence in Arabidopsis.

### 2.3. ERD7 Is Involved in Leaf Senescence Induced by ABA and NaCl

Our data revealed that *ERD7* was strongly induced by different stress stimuli and leaf senescence, suggesting that ERD7 is likely involved in the stress-induced senescence process. To test this hypothesis, ABA and salt treatments were applied to detached leaves from Col-0, *ERD7* overexpression, and erd7 mutant plants, and the stress-induced senescence phenotypes were observed. First, the detached leaves of 5-week-old plants without senescence symptom were treated with 5 μM ABA for 5 days. As shown in Figure 3A, the *ERD7* overexpression leaves showed strong senescence symptoms, whereas the senescence phenotypes of erd7 mutant leaves were not obvious when compared with those of Col-0. Furthermore, the erd7 mutant leaves had a significantly higher and the *ERD7* overexpression leaves had a significantly lower chlorophyll content when compared with that of Col-0 (Figure 3B). Additionally, compared with Col-0, the detached leaves of *ERD7* overexpression lines showed a significantly higher whereas *erd7* mutant lines showed a significantly lower ion leakage rate (Figure 3C). The results suggest that ERD7 functions in promoting leaf senescence induced by ABA.

Next, we studied the role of ERD7 in NaCl-induced leaf senescence. The detached leaves of 5-week-old Col-0, *ERD7* overexpression, and *erd7* mutant lines were made to float in a treatment buffer containing 100 mM NaCl. After 5 days of treatments, the Col-0 leaves turned yellow, while the leaves from *erd7* mutant lines were minimally influenced with limited senescence symptoms. Meanwhile, the *ERD7*-overexpressing leaves displayed stronger senescence symptoms (Figure 3D). By measuring the chlorophyll content and the ion leakage rate of the detached leaves, it was shown that the *ERD7*-overexpressing leaves had lower chlorophyll contents and higher ion leakage rates. On the contrary, erd7 mutant leaves had a significantly higher chlorophyll content but a lower ion leakage when compared with those of Col-0 (Figure 3E,F). These results indicate that ERD7 might be involved in regulating age-dependent and stress-induced leaf senescence processes.

### 2.4. The Phosphorylation Modification of Thr-225 and Ser-262 Residues Is Up-Regulated during Leaf Senescence

**Figure 4:**
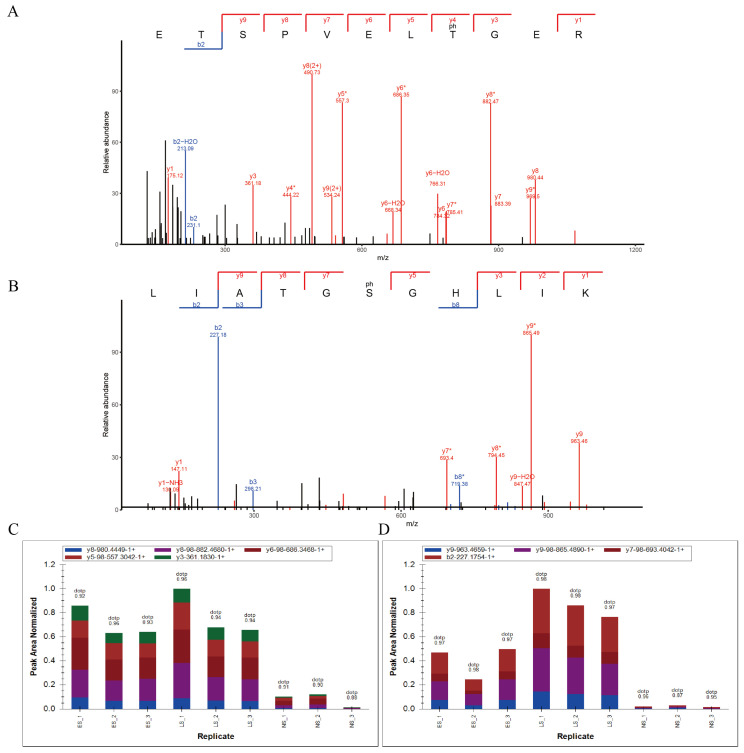
The phosphorylation modification of Thr-225 and Ser-262 residues is up-regulated during leaf senescence. (**A**) MS/MS spectrum of the representative peptide. The representative peptide ETSPVELT(ph)GER derived from ERD7 protein. The observed b- and y-ions were labeled in the MS/MS spectrum. T(ph) = phosphorylated amino acid residue. “*” is used to indicate where phosphorylation occurs. (**B**) MS/MS spectrum of the representative peptide. The representative peptide LIATGS(ph)GHLIK derived from ERD7 protein. The observed b- and y-ions were labeled in the MS/MS spectrum. T(ph) = phosphorylated amino acid residue. “*” is used to indicate where phosphorylation occurs. (**C**) quantification of phosphopeptide ETSPVELTGER by integration of fragment ion. (**D**) quantification of phosphopeptide LIATGSGHLIK by integration of fragment ion.

We performed quantitative phosphoproteomics studies during leaf senescence using TMT labeling and phosphorylation enrichment techniques and a quantitative proteomics study strategy using high-resolution liquid chromatography–mass spectrometry. After immunoprecipitation and trypsin digestion, 36 total spectra for ERD7 in NS, ES, and LS samples were identified using LC-MS/MS analysis. Then, a total of 15 peptides were identified with spectrogram analysis, and the percentage of identified peptide sequences covering the protein sequence (Coverage) was 49.2%, in which two phosphopeptides of ERD7 (Protein Accession: A0A1P8AYB1) were identified with mass errors of 0.13961 and 0.75399. The ETSPVELT(ph)GER (*m*/*z* = 649.287215) and LIATGS(ph)GHLIK (*m*/*z* = 397.2162) peptides were derived from ERD7 (Table S1). Typical fragment ions correspond to cleavages along the peptide backbone. In this context, the well-defined series of b-ion and y-ion correspond to the cleavage of different peptide bonds within the sequences of the ETSPVELT(ph)GER and LIATGS(ph)GHLIK peptides, specifically (b2; y1, y3, y4, y5, y6, y7, y8, and y9) for ETSPVELT(ph)GER (Figure 4A) and for LIATGS(ph)GHLIK (b2, b3, and b8; y1, y2, y3, y5, y7, y8 and y9) (Figure 4B). The phosphorylation sites were located at the amino acid residues Thr-225 and Ser-262, respectively, and the phosphorylation levels were significantly up-regulated during senescence. To validate the reliability of the phosphoproteomics data, the phosphorylation levels of the candidate phosphopeptides were further quantified using the Parallel Reaction Monitoring (PRM) strategy in this study (Figure 4C,D). The results showed that the NS/ES and NE/LS ratio of phosphopeptide ETSPVELT(ph)GER was 0.10874 and 0.09919, and the *p* value was 0.00147 and 0.00373, respectively. Meanwhile, the NS/ES and NE/LS ratio of phosphopeptide LIATGS(ph)GHLIK was 0.05846 and 0.02689, and the *p* value was 0.00889 and 0.00024 (Table 1), suggesting that the phosphorylation modification of Thr-225 and Ser-262 were significantly up-regulated during leaf senescence. The results provide clues that the phosphorylation modification of Thr-225 and Ser-262 residues could play important roles in mediating ERD7’s function in leaf senescence regulation.

### 2.5. The Phosphorylation State of Thr-225 and Ser-262 Residues Is Critical for the Function of ERD7 in Regulating Leaf Senescence

**Figure 5:**
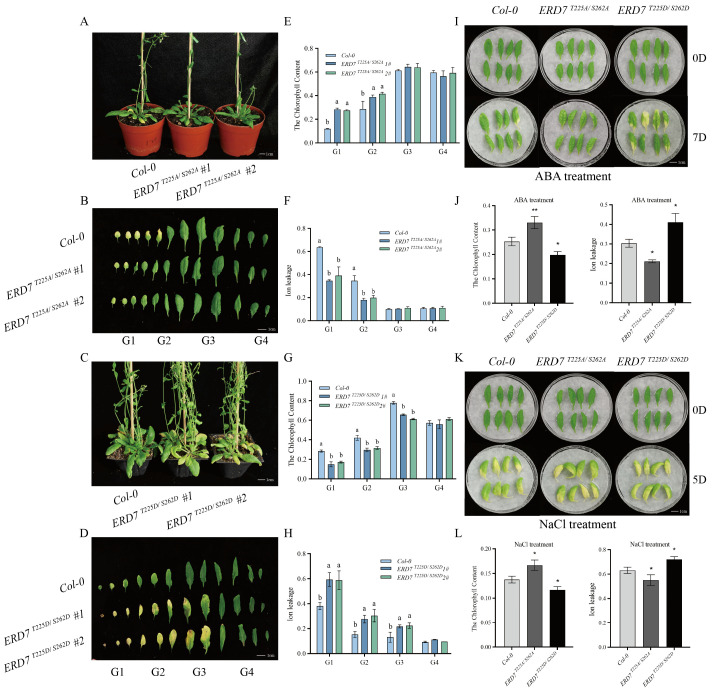
T225 and S262 residues affected the function of ERD7 in leaf senescence regulation. (**A**) Leaf phenotypes of 6-week-old Col-0 and ERD7^T225A/S262A^ transgenic plants. (**B**) Phenotypes of 12 rosette leaves of Col-0 and ERD7^T225A/S262A^ transgenic plants. (**C**) Leaf phenotypes of 6-week-old Col-0 and ERD7^T225D/S262D^ transgenic plants. (**D**) Phenotype of 12 rosette leaves of Col-0 and ERD7 ^T225D/S262D^ transgenic plants. (**E**,**F**) Measurement of chlorophyll content and ion leakage in 12 rosette leaves of different strains. (**G**,**H**) Measurement of chlorophyll content and ion leakage in 12 rosette leaves of different strains. (**I**) Phenotypes of isolated leaves of Col-0, ERD7^T225A/S262A^, and ERD7^T225D/S262D^ transgenic plants after 5 μM ABA. (**J**) Chlorophyll content and ion leakage measurements of detached leaves of different lines as indicated after 7 d of ABA treatment. (**K**) Phenotypes of isolated leaves of Col-0, ERD7^T225A/S262A^, and ERD7^T225D/S262D^ transgenic plants after 100 mM NaCl treatments. (**L**) Chlorophyll content and ion leakage measurements of detached leaves of different lines as indicated after 5 d of salt stress treatment. Asterisks indicate statistically significant differences (* *p* < 0.05; ** *p* < 0.01) using a Student’s *t*-test. Different letters above columns indicate significant differences based on Duncan’s multiple range test (*p* < 0.05). Three independent experiments were performed.

To study the significance of T225 and S262 phosphorylation residues on ERD7 protein function, we first investigated the effect of these residues in subcellular localization of ERD7. We generated *35S*:: ERD7^T225A/S262A^-GFP and *35S*:: ERD7^T225D/S262D^-GFP constructs, in which both T225 and S262 phosphorylation residues were mutated to alanine (A) to mimic a state of sustained inactivation of phosphorylation or to aspartic acid (D) to mimic a state of hyperphosphorylation at the site, respectively. Then, the constructs were individually transiently expressed in the leaf of *Nicotiana benthamiana* to detect the ERD7 subcellular localization. The results showed that ERD7, ERD7^T225A/S262A^, and ERD7^T225D/S262D^ were all localized to the cell membrane, suggesting that both T225 and S262 residues were not critical for ERD7 subcellular localization (Figure S3). To further examine whether both T225 and S262 residues were necessary for ERD7s function in age-dependent and stress-induced leaf senescence, we obtained *35S*:: ERD7^T225A/S262A^ and *35S*:: ERD7^T225D/S262D^ transgenic plants. Four independent lines named ERD7^T225A/S262A^#1, ERD7^T225A/S262A^#2, ERD7^T225D/S262D^#1, and ERD7^T225D/S262D^-2# were selected for further study, respectively. The results showed that ERD7^T225A/S262A^#1 and ERD7^T225A/S262A^#2 plants exhibited a delayed leaf senescence compared to that of Col-0 (Figure 5A,B). In contrast, both ERD7^T225D/S262D^#1 and ERD7^T225D/S262D^#2 exhibited a premature leaf senescence when compared with that of Col-0 (Figure 5C,D). The visible senescence phenotypes were also supported by the measurement of the chlorophyll content and the ion leakage. Compared to the wild type, the ERD7^T225A/S262A^#1 and ERD7^T225A/S262A^#2 plants exhibited a significantly higher chlorophyll content (Figure 5E) but lower ion leakage rates (Figure 5F) in G1 and G2 leaf groups. In contrast, when the residues of T225 and S262 were mutated to D225 and D262, the transgenic plants displayed decreased photosynthetic activities (Figure 5G) and increased membrane ion leakage (Figure 5H) in G1 and G2 leaf groups. These results suggested that ERD7 regulates leaf senescence potentially through the phosphorylation of the two residues, T225 and S262.

We further confirmed that both T225 and S262 were also critical in stress-induced leaf senescence. The 6th detached leaves of 5-week-old Col-0 and ERD7^T225A/S262A^#1 and ERD7^T225D/S262D^#1 plants were treated in 5 μM ABA and 100 mM NaCl solutions, respectively. The results showed that the leaves of ERD7^T225D/S262D^#1 exhibited accelerated yellowing after 7-day treatment of 5 μM ABA compared with those of the wild type, whereas the detached leaves of ERD7^T225A/S262A^#1 remained green (Figure 5I). Additionally, all leaves turned yellow in 5 days with 100 mM NaCl treatments, but the detached leaves of ERD7^T225D/S262D^#1 displayed much more severe leaf senescence symptoms. On the other hand, ERD7^T225A/S262A^#1 leaves showed a delayed senescence when compared with that of Col-0 (Figure 5K). Additionally, the chlorophyll content and the ion leakage rate were measured, and the results were consistent with the visible phenotypes (Figure 5J,L). Together, these results suggest that T225 and S262 are critical for the function of ERD7 in the regulation of age-dependent and stress-induced leaf senescence.

### 2.6. ERD7 Regulates Hydrogen Peroxide Accumulation Depending on T225 and S262 Phosphorylation

**Figure 6:**
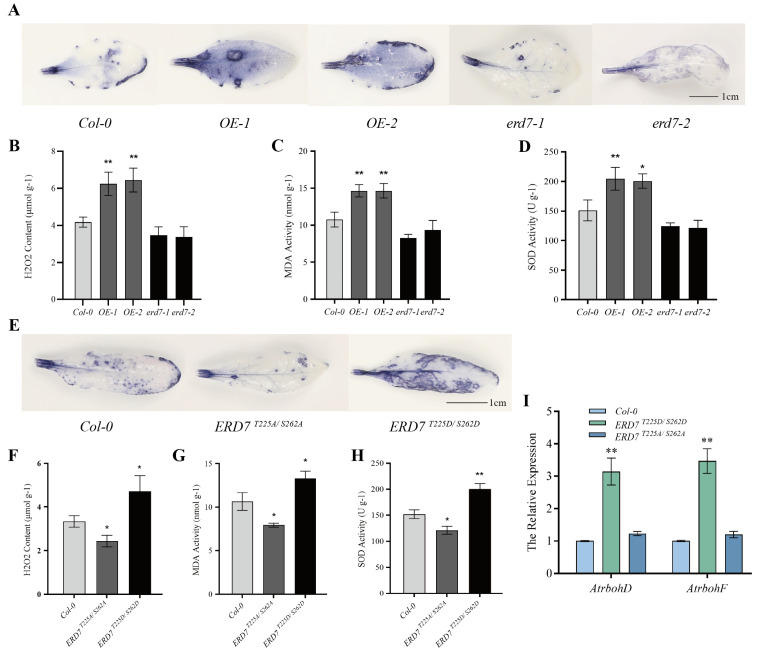
ERD7 regulated the ROS production depending on the phosphorylation modification of T225 and S262 residues. (**A**) NBT staining of Col-0, *ERD7* overexpression, and mutant plants. (**B**–**D**) Endogenous H_2_O_2_, MDA, and SOD contents in 5-week-old Col-0, *ERD7* overexpression, and mutant plants. (**E**) NBT staining of Col-0, ERD7^T225A/S262A^, and ERD7^T225D/S262D^ transgenic plants. (**F**–**H**) Contents of endogenous H_2_O_2_, MDA, and SOD in 5-week-old Col-0, ERD7 ^T225A/S262A^, and ERD7 ^T225D/S262D^ transgenic plants. (**I**) Expression of *AtrbohD* and *AtrbohF* within Col-0, ERD7^T225A/S262A^, and ERD7 ^T225D/S262D^ transgenic plants. Asterisks indicate statistically significant differences (* *p* < 0.05; ** *p* < 0.01) using a Student’s *t*-test. Three independent experiments were performed.

ROS are recognized as important signals during leaf senescence and stress responses [34]. To gain insight into the mechanism of ERD7 in leaf senescence regulation, we tested whether ERD7 was involved in hydrogen peroxide accumulation. Firstly, we examined H_2_O_2_ levels in plants with different genotypes grown for 42 days with nitroblue tetrazolium (NBT) staining. The results revealed that *erd7* mutants accumulated less H_2_O_2_, whereas *ERD7* overexpressing leaves accumulated significantly more H_2_O_2_ compared with that of Col-0 (Figure 6A), which was consistent with the results of H_2_O_2_ quantification assays (Figure 6B). Furthermore, malondialdehyde (MDA) and SOD production, indicators of oxidative attack on membrane lipids and membrane injury, respectively, were significantly lower in *erd7* mutants but higher in *ERD7* overexpressing lines compared with Col-0 (Figure 6C,D). These results suggested that ERD7 was involved in regulating hydrogen peroxide accumulation.

Given that both T225 and S262 residues were critical for the function of ERD7, we also determined the H_2_O_2_ levels in Col-0, ERD7 ^T225A/S262A^, and ERD7 ^T225D/S262D^ plants. NBT staining and H2O2 quantification results demonstrated that ERD7 ^T225D/S262D^#1 displayed an intensive dark blue color and significantly higher ROS levels, but ERD7 ^T225A/S262A^#1 showed a lighter color and lower ROS levels compared with Col-0. Moreover, MDA and SOD production was consistent with the ROS levels (Figure 6G,H). Thus, T225 and S262 could play important roles in ERD7’s regulation of ROS homeostasis.

Next, we asked the question of how T225 and S262 residues affect the function of ERD7? It is reported that the NADPH oxidase-mediated pathway is the most important pathway for ROS production [35]. In Arabidopsis, two NADPH oxidase-encoding genes, AtrbohD and AtrbohF, have been shown to play key roles in the ABA-mediated ROS production [36]. The qRT-PCR analysis showed that *AtrbohD* and *AtrbohF* transcripts were significantly up-regulated in ERD7^T225D/S262D^#1 plants compared with those of Col-0 (Figure 6I). Together, these results suggested that T225 and S262 residues, as potential senescence-dependent phosphorylated sites, might be involved in the ERD7 regulation of ROS accumulation through the modulation of the expression of *AtrbohD* and *AtrbohF*.

## 3. Discussion

As the most important organ for storing and producing energy in plants, the proper senescence timing of leaves is crucial for plant development and productivity [37]. As a complex and orderly controlled biological process, although leaf senescence can be induced by a variety of factors, the senescence process is strictly regulated by genes [38]. Additionally, plants are constantly exposed to environmental changes that affect their performance. In this study, age-dependent and stress-induced senescence were examined in the *erd7* mutant and *ERD7* overexpression transgenic plants at the phenotypical and physiological levels. We show that the *erd7* mutation increased leaf longevity by delaying the onset of senescence during age-dependent natural senescence and stress-induced senescence. Our findings suggest that ERD7 might be a hub of crosstalk between age-dependent and stress-induced leaf senescence. Interestingly, we demonstrated that the phosphorylation modification of Thr225 and Ser262 were significantly up-regulated during leaf senescence. Moreover, these senescence-responsive phosphopeptides play critical roles in the function of ERD7 for regulating senescence. 

In the natural environment, the growth and development of plants suffer all the time from the challenges of unfavorable environment. To adapt to these stresses, plants developed various defense mechanisms including changing the plant senescence process. Generally, stresses accelerate older leaf senescence, leading to the removal of injured tissues and the delivery of nutrients to younger tissues to improve adaptability [39]. Interestingly, many stress-related genes might also be involved in plant senescence process. To date, extensive studies have revealed that a wide range of transcription factors, including WRKY and NAC, are involved in the regulation of age-dependent as well as stress-induced leaf senescence [40,41,42]. For example, WRKY54 and WRKY70 act synergistically as the negative regulators of leaf senescence in Arabidopsis, participating in a regulatory network integrating internal and environmental cues to regulate the onset and progression of leaf senescence [43]. OsNAC2, which is known to negatively regulate multiple abiotic stress tolerances, also promotes leaf senescence in rice [44]. TaNAC29 plays a crucial role in senescence and abiotic stress responses [45]. It is hypothesized that these genes play important roles in the signaling pathways of stress response and age-dependent senescence, and that ERD genes are dehydration-induced early response genes in *Arabidopsis thaliana*, consisting of 16 members [27]. However, to date, only a few ERD members have undergone functional characterization. In this study, ERD7 was identified as a positive regulator of leaf senescence in Arabidopsis, with overexpressed transgenic plants exhibiting premature senescence. Conversely, the *erd7* mutant effectively delayed leaf senescence. Previous studies have demonstrated the involvement of ERD members in senescence, playing a pivotal role in stress response. For instance, in Arabidopsis, ERD1 encodes a protein induced by dehydration and up-regulated during natural and dark-induced senescence [46,47]. ERD4 can be significantly induced under salt stress treatment, enhancing plant tolerance to abiotic stress and contributing to the early stages of plant adaptation to adversity [48]. ERD10 and Response to Desiccation 29A (RD29A) activate the expression of ERF34, identified as a negative regulator of salt stress-induced leaf senescence, thereby integrating salt stress signaling with the leaf senescence program [49]. Furthermore, in this study, *ERD7* was found to be induced under various stress treatments such as low temperature, NaCl, and ABA. Current studies on ERD7 indicate it as a marker corresponding to abiotic stress in plants [50,51], with a strong up-regulation in response to both biotic and abiotic stresses, including drought, dehydration, cold, salt, etc. [23,27,52]. Additionally, ERD7 is capable of remodeling cell membrane lipids during cold stress in *Arabidopsis thaliana* [53]. These findings suggest the significance of ERD7 in plant stress response and development. However, the functional analysis of ERD7 in the regulation of leaf senescence in *Arabidopsis thaliana* remains ambiguous, particularly its intricate involvement in age-dependent and stress-induced leaf senescence. Intriguingly, prior reports have indicated the enrichment of ERD7 in lipid droplets (LDs) based on the proteomic analysis of senescent leaves in Arabidopsis. It was noted that the C-terminal senescence structural domain of the protein was both necessary and sufficient for LD targeting [25,54]. This observation prompts a reevaluation of ERD7s role, suggesting a potential link between ERD7 and LDs under stress conditions. This novel perspective provides a valuable avenue for deeper investigations into the mechanism by which ERD7 participates in the regulation of leaf senescence in Arabidopsis. Moreover, ERD7 may serve as a nexus between age-dependent senescence and responses to environmental stimuli, such as ABA and salt stress, co-regulating leaf senescence in Arabidopsis. Thus, beyond its role as a marker for plant stress response, ERD genes, including *ERD7*, may exert a crucial influence on the regulation of leaf senescence [24]. Our ongoing studies on the role of ERD7 in age-dependent and stress-induced leaf senescence are poised to significantly enhance our understanding of ERD gene functions and explore their potential contributions to crop improvement, particularly in the context of stress-induced senescence.

Protein phosphorylation can be used as a molecular switch to regulate different hormone signaling pathways and stress tolerance in plants [55]. Phosphorylation often plays an important regulatory role in the normal performance of protein function including protein localization, protein–protein interactions, and cellular signaling [56]. An increasing number of studies have confirmed that protein phosphorylation plays a role in leaf senescence regulation [57]. Protein kinases that catalyze protein phosphorylation were reported to be involved in leaf senescence. For example, Receptor protein kinase1 (RPK1) affects ABA-induced leaf senescence [58]; ENHANCED DISEASE RESISTANCE1 functions as a negative regulator of ethylene-induced senescence [59]; mitogen-activated protein kinase cascade involving MPK9 and MPK6 plays a positive role in regulating leaf senescence [60]; Arabidopsis Histiding Kinase3 functions in mediating the anti-senescence effect of cytokinins through the phosphorylation of ARABIDOPSIS RESPONSE REGULATOR2 [61]. Although several studies have confirmed that protein phosphatase plays a critical role in leaf senescence regulation, the mechanisms underlying how phosphorylation modification participates in leaf senescence are not well understood. In this study, based on the results of LC-MS/MS and PRM quantitative analyses, we found that the phosphorylation modification levels of two residues of ERD7, T225 and S262, were both significantly up-regulated during senescence using PRM strategy, a phosphorylation quantification method with superior selectivity and sensitivity. Furthermore, we found that the mutation of these sites severely affected the normal functioning of ERD7, providing the direct evidence that the two phosphorylation sites, T225 and S262, play essential roles in age-dependent and stress-induced leaf senescence. 

ROS are the ubiquitous components of plant signaling pathways that control a wide range of physiological functions including leaf senescence [11] and stress responses [17]. In plants, plasma membrane-localized NAPDH oxidases, known as respiratory burst oxidase homologs (RBOHs), are responsible for provoking ROS bursts [62]. There are 10 RBOHs (AtrbohA-J) in Arabidopsis [63], in which AtrbohD and AtrbohF are responsible for promoting ROS production in response to biotic and abiotic stresses [64,65]. In this study, we first examined the ROS levels in different genotypical Arabidopsis lines using NBT chemical staining and the quantification of H_2_O_2_, MDA, and SOD. The results showed that ERD7^T225D/S262D^ enhanced the ROS accumulation, meanwhile, the expression of *AtrbohD* and *AtrbohF* was significantly up-regulated in the same lines. The results strongly suggested that ERD7, regulating age-dependent and stress-induced leaf senescence mediated by ROS production, relied on the phosphorylation modification of Thr-225 and Ser-262 residues. 

## 4. Materials and Methods

### 4.1. Plant Materials and Growth Conditions

Arabidopsis seeds were first sterilized with 75% alcohol for 5 min, and then washed with anhydrous ethanol for 1 min. The seeds were germinated and grown on 1/2 MS media in a plant growth chamber (Conviron, Winnipeg, ManitobaCanada). At the stage of two true leaves, the *Arabidopsis* seedlings were transferred to sterilized soil for further cultivation, and the temperature of the incubation room was 22–24 °C, with 16 h of light and 8 h of darkness, and the relative humidity was between 65% and 70%, with a light intensity of 150 μmol/M2/S. The mutants *erd7-1* (CS436725) and *erd7-2* (N864658) used in this study were obtained from the Arabidopsis Biological Resource Center (ABRC) and AraShare (www.arashare.cn (accessed on 25 February 2020)), respectively.

### 4.2. Construct Generation and Arabidopsis Transformation

To generate the *ERD7* overexpressing constructs, the full length of *ERD7* CDS was amplified with PCR method using specific primers (Table S2). According to the instructions of the Invitrogen gateway kit (kit No.11789 (BP Clonase); No.117910 (LR Clonase), the PCR products were cloned into pDnor-207 vector using the BP enzyme. Subsequently, *ERD7* CDS was sub-cloned into pEarleyGate202 using the LR enzyme to form the *35S*:: *ERD7* construct. Then, the recombinant vector was transformed into *Agrobacterium tumefaciens* GV3101 and then transformed into *Arabidopsis thaliana* (Col-0) via the floral dip method (Clough and Bent, 1998). Transgenic plants were selected by glyphosate resistance. The T3 homozygous lines were used for senescence and stress treatments.

### 4.3. Chlorophyll, Fv/Fm, and Electrolyte (Ion) Leakage Rate Measurements

The determination of the chlorophyll content was conducted as previously described in [66]. The isolated leaves were soaked in 96% (*v*/*v*) ethanol (3–4 mg of tissue in 1 mL of ethanol) in dark until chlorophyll was completely dissolved. The total chlorophyll content was quantified by measuring absorbance at 646 and 663 nm. Total chlorophyll = 17.76 (A646) + 7.34 (A663). To measure the ion leakage rate of *Arabidopsis* leaves, the leaves were immersed in deionized water and slowly shaken at 25 °C for 30 min, and the conductivity of the aqueous solution was measured by using a benchtop conductivity meter (Leici, DDS-11A, INESA Instrument, Shanghai, China). The aqueous solution was heated and boiled continuously for 10 min, and the conductivity of the aqueous solution was measured again, and the percentage of the conductivity was calculated based on the previous and the latter measurements. The percentage of conductivity between the two measurements is the ion leakage rate of the leaves.

### 4.4. Induction of Senescence by Detached Leaf Treatments

The treatments were performed as previously described [67]. Briefly, the 6th and 7th rosette leaves were isolated from 5-week-old plants and made to float on Petri dishes under the treatments of 5 μM ABA or 100 mM NaCl. All treatments were carried out under continuous light at 22 °C for 5–7 days.

### 4.5. Phytohormone and Stress Treatments

The 7th and 8th rosette leaves were detached from plants grown for 5 weeks under continuous light conditions for hormone and abiotic stress treatments. The leaves were collected after 0, 1, 5 and 7 h of treatments. For hormone treatments, leaves were transferred to liquid media containing the phytohormones ABA (1 mM), SA (20 mM), IAA (20 mM), MeJA (20 mM), or ACC (20 mM). For stress treatments, the leaves were submerged in a salt solution at 100 mM NaCl or 200 mM mannitol. Cold treatments were performed by incubating leaves at 4 °C.

### 4.6. ROS Detection Experiments

Plant tissues produce a variety of reactive oxygen species (ROS) under stressful environmental conditions, and superoxide anion is a type of reactive oxygen species that reduces NBT (nitrogen blue tetrazolium), showing blue to dark blue coloration after staining in areas with superoxide anion aggregates. For NBT staining, the 6th and 7th rosette leaves of 5-week-old plants were taken and soaked in NBT staining buffer (0.5 mg/mL NBT in 10 mM potassium phosphate buffer, pH 7.6) overnight. Leaf chlorophyll was removed in a fixative (ethanol/acetic acid/glycerol = 3:1:1) and then stored in ethanol/glycerol (4:1) solution until photographs were taken. Endogenous H_2_O_2_, MDA, and SOD contents were determined according to the manufacturer’s (Beijing Solarbio Science & Technology Co., Ltd., Beijing, China) instructions. Three biological replicates were performed.

### 4.7. Quantitative RT-PCR Assays

The total RNA was isolated from frozen leaf samples using trizol (CW biotech, Beijing, China) according to the instructions of the manufacturer. First-strand cDNAs were generated from the total RNA (1 µg) with reverse transcription using HiScript II 1st Strand cDNA Synthesis Kit (Vazyme, Nanjing, China). The quantitative RT-PCR assays were performed on an ABI 7500 (ABI, Los Angeles, CA, USA) using ChamQ Universal SYBR qPCR Master Mix (Vazyme, Nanjing, China). The relative expression levels of genes were calculated using the 2^−ΔΔCt^ method. *Actin2* was applied as an internal reference gene to normalize all data. All qRT-PCRs were performed with three biological replications. Primer sequences are listed in Table S2.

## Figures and Tables

**Table 1:** The quantitative results of protein phosphorylation with PRM.

Protein Accession	Peptide Modified Sequence	NS/ES Ratio	NS/ES *p* Value	NS/LS Ratio	NS/LS *p* Value
A0A1P8AYB1	ETSPVELT(ph)GER	0.10874	0.00147	0.09919	0.00373
A0A1P8AYB1	LIATGS(ph)GHLIK	0.05846	0.00889	0.02689	0.00024

## Data Availability

The authors confirm that the data supporting the results of this study are available in the article and Supplementary Materials.

## Supplementary Materials

The following supporting information can be downloaded at: https://www.mdpi.com/article/10.3390/ijms25021328/s1.
