# Supplementary material for: Phosphorylation of Thr-225 and Ser-262 on ERD7 Promotes Age-Dependent and Stress-Induced Leaf Senescence through the Regulation of Hydrogen Peroxide Accumulation in Arabidopsis thaliana
Source: Int J Mol Sci. 2024 Jan 22;25(2):1328. doi: 10.3390/ijms25021328 (PMC10815956; doi:10.3390/ijms25021328)
Supplement: Supplementary file 1 [file ijms-25-01328-s001.zip › ijms-2840242-supplementary.pdf]

Supplementary Figures

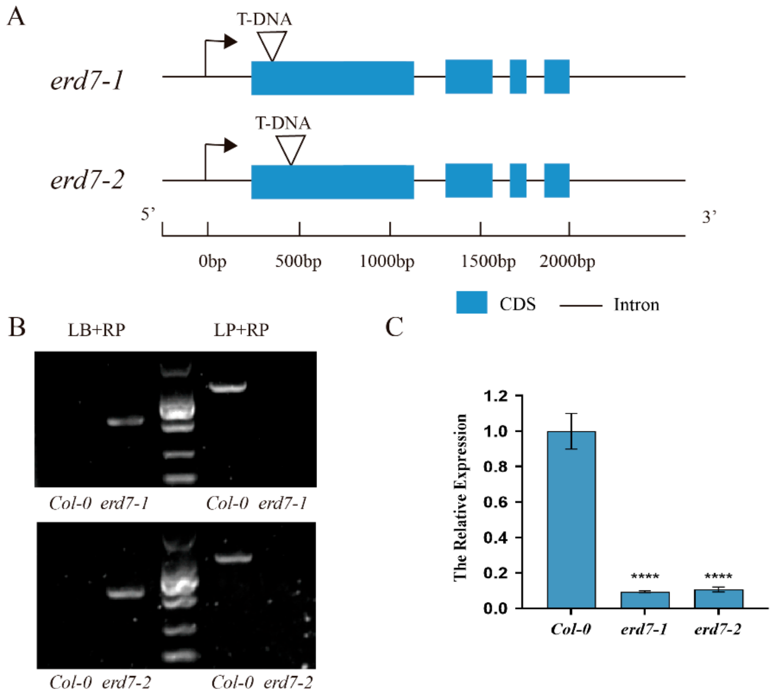

Figure S1. Generation and characterization of ERD7 transgenic Arabidopsis mutant lines. (A) Schematic representation of the gene structure and T-DNA insertion of the two mutant lines of ERD7 (B) Verification of purity of erd7 mutant by triple priming method (C) The relative expression levels of ERD7 in Mutant lines.

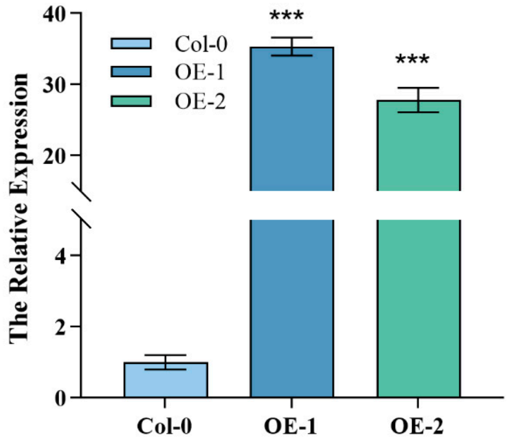

Figure S2. The relative expression levels of ERD7 in overexpression lines.

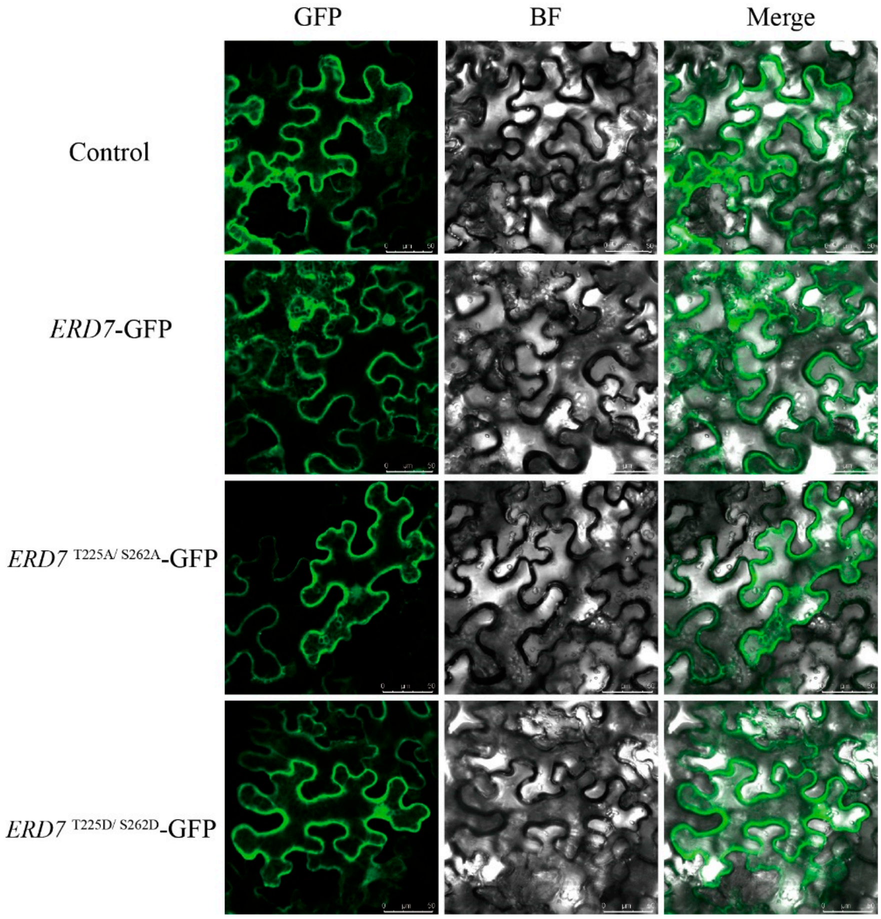

Figure S3. Subcellular localization analysis of ERD7 and ERD7 phosphorylation sites after mutation.

Table S1. Identification of ERD7 protein phosphorylation sites by mass spectrometry

Gene name	Position	Amino acid	Score	Modified sequence	Modifications	m/z	Mass error [ppm]	Score
ERD7	225	T	100.69	ETSPVELT(1)GER	Phospho (STY)	649.287215	0.61419	130.78
ERD7	262	S	130.78	LIAT(0.01)GS(0.99)GHLIK	Phospho (STY)	397.2162	1.1773	121.56

Table S2. Primers used in this study.

Primer Name	Sequence (5'-3')
AtrbohD-Q-F	TCCACGCACTCAAAGGTCTC
AtrbohD-Q-R	CCGGCGAGCTAATGCTCTAA
AtrbohF-Q-F	TGACACGCCAAGACGAAAGA
AtrbohF-Q-R	GAGCAGAACGAGCATCACCT
erd7-1-LP	ACTTCTTCTCTTTACCCTACTGT
erd7-1-RP	CCATTAGATCTGCAGCTTCAC
erd7-2-LP	ACTTCTTCTCTTTACCCTACTGT
erd7-2-RP	CCATTAGATCTGCAGCTTCAC
LB	GTCCGCAATGTGTTATTAAGTTG
ERD7-Q-F	CACAAGTATGGTGGTAAAGCGG
ERD7-Q-R	AGCTGCAGATCTAATGGCGG

SAG12-Q-F	TCCAATTCTATTCGTCTGGTGTGT
SAG12-Q-R	CCACTTTCTCCCCATTTTGTTC
ACT2-Q-F	TGTGCCAATCTACGAGGGTTT
ACT2-Q-R	TTTCCCGCTCTGCTGTTGT
PRI101-ERD7-F	CGACCCCGGGGGTACCATGGAAT
PRI101-ERD7-R	CTTGCTCACCATGAATTCCTAGGCT
